# Total Anterior Uncinatectomy During Anterior Discectomy and Fusion for Recurrent Cervical Radiculopathy: A Two-dimensional Operative Video and Technical Report

**DOI:** 10.7759/cureus.7466

**Published:** 2020-03-30

**Authors:** Fidel Valero-Moreno, William Clifton, Aaron Damon, Mark Pichelmann

**Affiliations:** 1 Neurological Surgery, Mayo Clinic, Jacksonville, USA

**Keywords:** uncinate, uncinectomy, vertebral artery, anterior cervical discectomy and fusion, spine surgery, cervical spine, uncinatectomy, uncovertebral joint, surgical video, anatomy

## Abstract

A common cause of cervical radiculopathy from degenerative foraminal stenosis is severe uncovertebral hypertrophy. It is difficult to accomplish complete foraminal decompression in these cases with posterior techniques without the removal of a large portion of the facet joint. Total removal of the uncovertebral joint from an anterior approach allows for complete decompression of the exiting cervical nerve root and has been shown to be a safe technique. In this surgical video and technical report, we demonstrate the surgical anatomy and operative technique of a two-level anterior uncinatectomy during anterior discectomy and fusion (ACDF) for recurrent cervical radiculopathy after a previous multi-level posterior foraminotomy. The patient is a 67-year-old male with a progressive left arm and neck pain with radiographic, clinical, and electrophysiologic diagnostic evidence of active C6 and C7 radiculopathies from degenerative foraminal stenosis at the C5-6 and C6-7 levels. Posterior foraminotomies had been performed without significant improvement in his radicular pain. A repeat MRI demonstrated lateral foraminal stenosis from severe uncovertebral joint hypertrophy at the C5-6 and C6-7 levels. After acquiring informed consent from the patient, an anterior approach was performed with complete removal of the uncovertebral joints at both levels with discectomy and fusion. Postoperatively, the patient had complete resolution of his radicular pain and remained pain-free at the latest follow-up. Complete uncinatectomy and ACDF is an effective technique for complete foraminal decompression in cases of refractory radiculopathy and neck pain after unsuccessful posterior decompression.

## Introduction

Cervical radiculopathy is a common indication for neurosurgical consultation [1]. Anterior discectomy and fusion (ACDF) can be employed in cases of cervical foraminal stenosis from advanced degeneration, including uncovertebral joint hypertrophy [2,3]. In these cases, it is difficult to remove the offending bony compression using a posterior approach, i.e., foraminotomy, which can result in recurrent pain from residual compression lateral in the cervical foramen [4]. Total uncinatectomy can be performed safely for complete foraminal decompression using an anterior approach [5,6]. In this technical note, we present a case illustration and technical review of a two-level ACDF with total uncinatectomy for recurrent radiculopathy following a previous posterior foraminotomy.

## Technical report

The details of the patient history, imaging findings, and operative technique are shown in Video 1. It is an operative video demonstrating the preoperative history, workup, and detailed surgical procedure of a two-level total uncinatectomy and ACDF for a patient with symptomatic cervical radiculopathy and severe uncovertebral joint hypertrophy.

**Video 1:** Anterior cervical uncinatectomy during ACDF for foraminal stenosis ACDF: anterior discectomy and fusion

The patient is a 67-year-old male who presented to our institution with a history of left arm and neck pain. He reported that the pain typically started in his neck and shot down his left arm into his thumb and middle finger, especially when performing an activity and turning his head to the left. His physical exam revealed no weakness, normal reflexes, but showed a positive Spurling's sign with left head-turning. An MRI study of his cervical spine revealed left-sided C5-7 cervical foraminal stenosis, and neurophysiologic studies confirmed the presence of left C6 and C7 radiculopathies. The diagnosis of left C6 and C7 cervical foraminal stenosis with radiculopathy was discussed with the patient, and the options for treatment were discussed in detail including continued conservative management, a posterior left foraminal decompression from C5-7, and ACDF. At that time, the patient wished to avoid fusion and wanted to pursue decompressive options for his radiculopathy. Informed consent was obtained, and left-sided posterior C5-7 foraminotomies were performed. However, the patient continued to have radicular pain for over six months after his initial presentation, along with increased neck pain. A repeat MRI demonstrated adequate posterior decompression of the C5-6 and C6-7 foramen; however, there was continued anterolateral compression of the nerve root by uncovertebral joint hypertrophy at both levels. 

**Figure 1:**
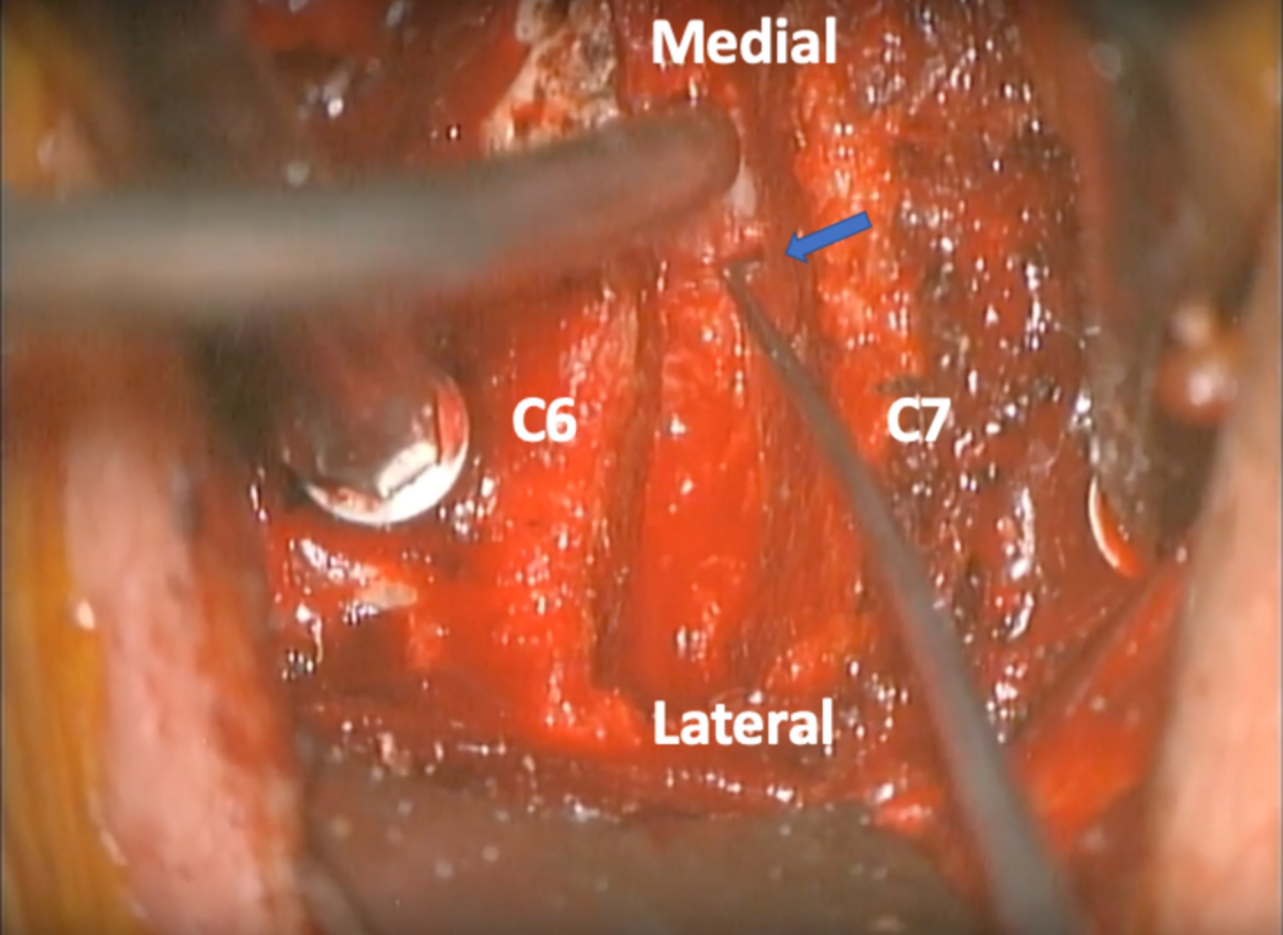
Operative view of the C6-7 disc space and left foramen After the uncinate is removed, the pedicle can be palpated with an angled instrument ensuring complete decompression of the foramen. The blue arrow indicates the location of the pedicle

Informed consent was acquired from the patient for a C5-7 ACDF following a thorough discussion of the risks, benefits, and alternatives of the surgery. The goal of the surgery was primarily to decompress his foramen and relieve the radiculopathy. The patient was brought to the operating room and placed in the supine position after the induction of general anesthesia. After a confirmatory pause, a localizing X-ray image was performed in order to plan the incision. The right side of the neck was prepped and draped in the usual fashion. A skin incision was made with a 10-blade knife, and monopolar cautery was used to identify the platysma muscle. The sternocleidomastoid muscle was identified, and a standard Smith-Robinson approach was used to gain access to the prevertebral space. The C5-6 vertebral level was localized using fluoroscopy, and Caspar pins were used at the C5 and C6 levels to distract the disc space. The annulus was incised and a high-speed burr was used to remove the disc and cartilaginous endplates. After the posterior longitudinal ligament was identified, the medial border of the uncinate was exposed using the burr. A freer dissector was then passed laterally to the uncinate process underneath the longus coli muscle, protecting the vertebral artery. The uncinate was then drilled until eggshell thin and then fractured medially into the disc space. The remaining portion of the uncovertebral joint articulating with the inferior portion of C5 was then gently dissected and the fibroligamentous attachment was removed. This final portion of the uncinate process was then removed, thus completely decompressing the foramen. The pedicle of C6 was palpated with a ball-tipped probe, ensuring complete removal of the offending uncinate (Figure 1).

**Figure 2:**
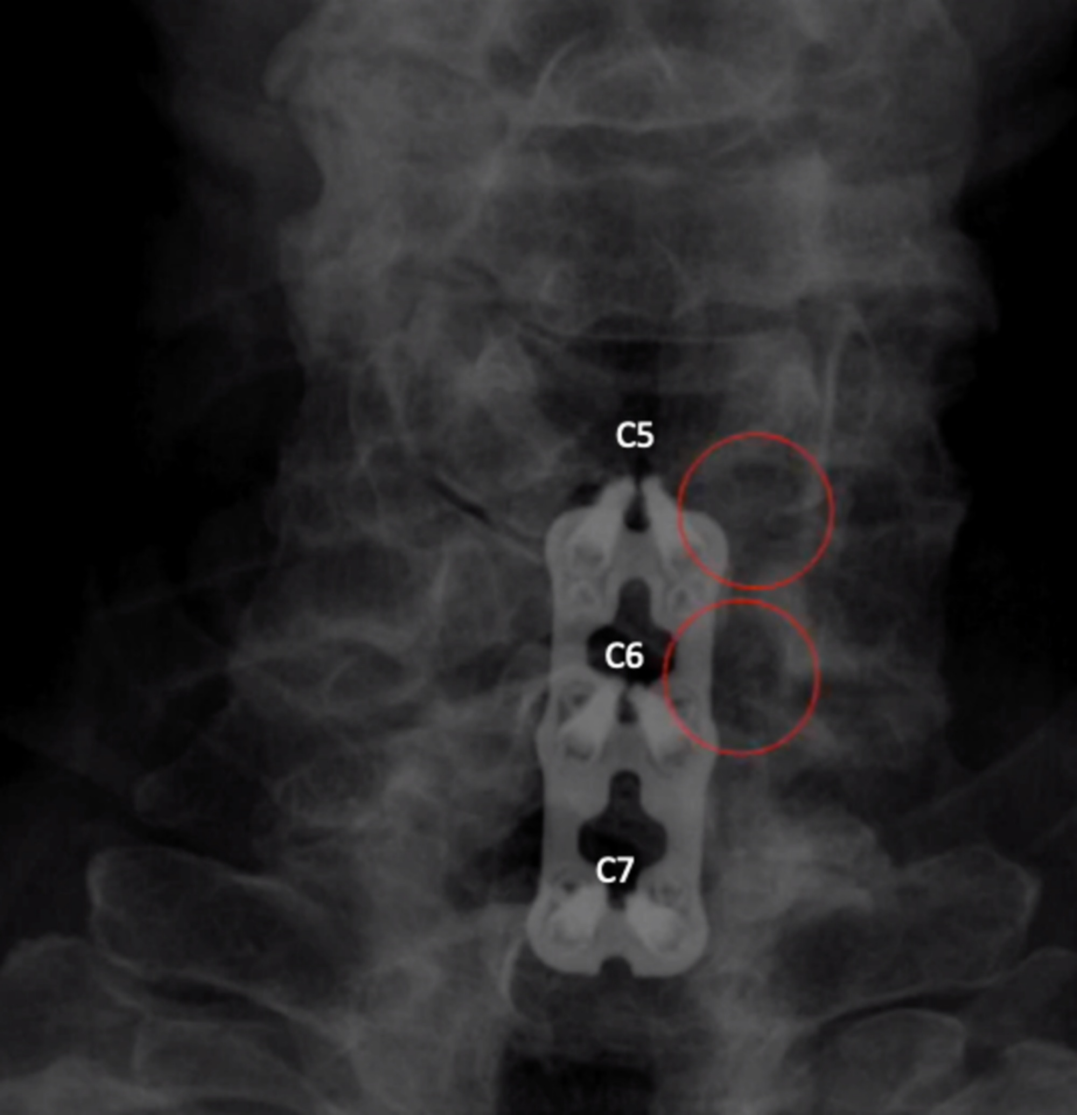
Postoperative X-ray after C5-7 ACDF with total uncinatectomies The image demonstrates good placement of hardware, with lucency in the C5-6 and C6-7 foramen (red circles) demonstrating complete removal of the uncinate and total decompression of the cervical roots ACDF: anterior discectomy and fusion

A disc space trial was placed, and an allograft disc graft was placed within the space. The Caspar pin at C5 was removed and then placed at C7. The same process of disc removal and uncinate drilling was performed at this level, completing the decompression of the C6-7 foramen. A graft was again placed into the C6-7 space. A plate and screws were placed in a good position, confirmed by an intraoperative X-ray (Figure 2). Postoperatively, the patient had complete resolution of his arm and neck pain, which remained stable at his latest follow-up.

## Discussion

Total uncinatectomy for cervical foraminal stenosis during ACDF is a useful procedure that provides total decompression of the cervical foramen. Uncinatectomy has historically been a controversial procedure, with many advocating for avoidance due to the risk of vertebral artery and nerve root injury [7,8]. There have been several proposed techniques for safe uncinate removal, including extensive lateral exposure of the vertebral artery, use of an osteotome, and use of an ultrasonic aspirator [9,10]. In our experience, we have found that the passage of a freer dissector lateral to the uncinate border provides adequate protection of the neurovascular structures of the cervical foramen. This technique provides a useful option for surgical treatment of radiculopathy caused by uncovertebral joint hypertrophy, especially in cases of failed posterior foraminotomy. Cervical foraminal stenosis caused by uncinate hypertrophy is difficult to treat with a posterior-only approach, as this technique does not address the cause of the stenosis that occurs laterally in the foramen, irritating the dorsal root ganglion and leading to continued pain [11,12]. Also, if the patient has central stenosis in addition to foraminal stenosis, total uncinatectomy combined with an ACDF can treat both pathologies with the same approach.

This case study illustrates the clinical relevance of the uncinate and uncovertebral joint in cervical radiculopathy caused by foraminal stenosis. The patient initially did not wish to undergo a fusion. In such cases, it is reasonable to try conservative therapy with injections or physical therapy in an attempt to see how much relief the patient gains before exploring surgical options. Purely decompressive procedures without fusion include posterior foraminotomy and anterior foraminotomy. Anterior foraminotomy can be performed through the same approach as an ACDF, but without significant disruption of the disc space, thus maintaining the stability of the motion segment at that level [9]. Posterior foraminotomy can be performed with good results; however, in cases of uncovertebral joint hypertrophy, it may be inadequate to completely decompress the entirety of the nerve root, as illustrated by this case. It is important as a surgeon to master all of these techniques in order to provide the best treatment options for patients with cervical foraminal disease, which often must be decided on a case-by-case basis.

## Conclusions

Total anterior uncinatectomy for failed posterior foraminotomy in patients with persistent radicular pain is a valid option for symptom relief. This can be performed safely with the use of a freer dissector lateral to the uncinate process with the protection of the vertebral artery; the uncinate can then be removed with a high-speed burr. A thorough knowledge of anatomy and the microsurgical relationships of the cervical foramen is necessary to provide the safest and most efficient care in this patient population.
